# Combined Bacopa, Phosphatidylserine, and Choline Protect Against Stress-Induced Neurotoxicity

**DOI:** 10.3390/biomedicines14020340

**Published:** 2026-02-01

**Authors:** Chiara Sasia, Giacomina Videtta, Nicoletta Galeotti

**Affiliations:** Department of Neurosciences, Psychology, Drug Research and Child Health (Neurofarba), Section of Pharmacology and Toxicology, Laboratory of Neuroinflammation and Cell Senescence, University of Florence, Viale G. Pieraccini 6, 50139 Florence, Italy; chiara.sasia@unifi.it (C.S.); giacomina.videtta@unifi.it (G.V.)

**Keywords:** *Bacopa monnieri*, phosphatidylserine, choline, stress, memory, microglia, neuroinflammation, neurotoxicity

## Abstract

**Background/Objectives**: Chronic stress leads to sustained elevations in cortisol levels, which promote neuronal damage and impair memory. Prolonged stress also enhances proinflammatory signaling. Adaptogens are plant-derived compounds associated with the ability to increase the body’s resistance to stress, thereby improving mental and physical performance. To identify potential interventions capable of attenuating stress-related memory alterations, this study investigated a formulation combining the adaptogen *Bacopa monnieri* L. with phosphatidylserine and choline (BPC). **Methods**: An in vitro model of stress-related neuroinflammation was established by exposing BV2 microglial cells to corticotropin-releasing hormone (CRH, 100 nM). SH-SY5Y cells exposed to conditioned medium from CRH-stimulated BV2 cells or to iron(II) sulfate and L-ascorbic acid (Fe/Asc) were used as models of neurotoxicity. **Results**: BPC attenuated CRH-induced proinflammatory microglial morphology, as well as the reduction in cell viability and cell number. BPC treatment restored the levels of stress-related markers, including SIRT-1, Nrf-2, and phosphorylated JNK (p-JNK). Furthermore, BPC protected against neurotoxicity induced by CRH and Fe/Asc and promoted cholinergic activation by restoring basal acetylcholinesterase (AChE) levels. The combined BPC formulation showed superior efficacy compared with its individual components across all experimental assays. **Conclusions:** Collectively, these findings indicate that the BPC formulation developed in this study effectively attenuates stress-related neuroinflammation and neurotoxicity. BPC may represent a promising strategy to help limit the progression of early cognitive dysfunction under conditions of prolonged stress.

## 1. Introduction

Stress is an inherent component of daily life and arises from a wide range of psychosocial demands. While acute stress can be adaptive, mobilizing energy and sharpening attention, chronic stress exerts deleterious effects on the brain and body. Prolonged activation of stress pathways disrupts neuroendocrine regulation, weakens immune defenses, and promotes vascular injury [1,2]. Growing evidence indicates that stress and memory are closely linked through shared neurobiological systems [3].

The physiological stress response involves the rapid activation of the sympathetic nervous system (SNS), followed by engagement of the hypothalamic–pituitary–adrenal (HPA) axis. Activation of these systems leads to the release of catecholamines, such as adrenaline, and glucocorticoids, primarily cortisol in humans [4,5]. These hormones exert widespread effects on brain regions that are critically involved in memory processing. While adrenaline does not cross the blood–brain barrier (BBB), cortisol readily penetrates the central nervous system (CNS) and binds to mineralocorticoid and glucocorticoid receptors located in the hippocampus, amygdala, and prefrontal cortex [6], brain regions central to memory, executive functioning, and attentional control. Cortisol enhances noradrenergic signaling in the basolateral amygdala and increases the responsiveness of this region to emotionally salient information [7]. Under chronic stress, HPA axis activity leads to sustained cortisol elevations that, consequently, damage neurons and can lead to measurable changes in memory performance. At the same time, SNS hyperactivity induced by high levels of adrenaline fosters hypertension and metabolic dysfunction [4].

Chronic stress profoundly alters immune regulation, shifting responses toward persistent inflammation. Although cortisol and catecholamines initially suppress immune activity, prolonged stress enhances proinflammatory signaling [8]. This leads to sustained microglial activation and the release of proinflammatory cytokines, promoting neuroinflammation and accelerating neurodegeneration [9].

Over recent decades, there has been growing scientific interest in understanding how stress affects cognitive functions, particularly memory. Numerous studies have demonstrated that enhancing endogenous antistress mechanisms can mitigate stress-induced neuropathological alterations [10,11]. In this context, natural compounds have gained increasing attention as adaptogenic strategies for the management of stress-related psychiatric and neurodegenerative disorders [12], largely due to their anti-neuroinflammatory properties [13]. Among them, *Bacopa monnieri* L., a plant with well-known antioxidant and anti-inflammatory properties, was traditionally used for memory and cognitive dysfunctions [14,15]. These beneficial effects were supported by several clinical studies [16,17,18]. Furthermore, attenuation of the stress reactivity of *B. monnieri* supplementation has been described [16,19].

In the effort to identify potential interventions to attenuate stress-related memory alterations, the present study investigated a combination of *B. monnieri* with phosphatidylserine and choline to improve efficacy. Phosphatidylserine is a glycerophospholipid with high concentrations in the brain. It plays a critical role in cellular membranes within the CNS supporting cellular functions by facilitating intercellular communication, receptor activation and electrical depolarization of neuronal membranes [20]. Phosphatidylserine has been proposed as supplementation for improving some memory functions [21]. Choline is a precursor of acetylcholine synthesis extensively used as a dietary supplement, with evidence indicating its effectiveness in supporting memory performance and improving cognitive function [22].

Our results indicated that combined *B. monnieri* extract, phosphatidylserine, and choline (BPC) suppressed the microglia proinflammatory morphology and restored cell viability in in a model of stress-correlated neuroinflammation by reducing the expression of stress-related markers. Furthermore, BPC exerted neuroprotective activities. An overall higher efficacy of BPC compared to single constituents was highlighted.

## 2. Materials and Methods

### 2.1. Reagents and Treatments

The model of stress-induced neuroinflammation was obtained by using corticotropin-releasing hormone (CRH, Merck, Milan, Italy) 100 nM as stimulus. Here, 1 mg of the fixed combination (BPC) consisting of a mixture of *Bacopa monneri* L. aerial part extract (Bacomind^TM^ [23]), containing 22% bacosides, phosphatidylserine, and choline bitartrate (Vitacholine^®^), kindly provided by BiosLine (Ponte San Nicolò, Italy), was dissolved in 1 mL of double-distilled H_2_O, thus obtaining a concentration of 1 mg/mL, which was then filtered to ensure sterility. Stock solutions were then diluted in complete RPMI medium to final concentrations of 0.1, 1, 10, and 100 µg/mL. Subsequently, individual constituents, choline (3.6 ng/mL), phosphatidylserine (2 ng/mL), and *B. monnieri* (1.25 ng/mL), were tested at concentrations present in 0.1 µg/mL of BPC, which was identified as the most effective dose.

### 2.2. Cell Culture

The BV2 murine microglial cell line (C57BL/6; Tema Ricerca, Genova, Italy; 16–20 passages) was used for in vitro experiments. Cells were cultured in 75 cm^2^ flasks containing RPMI 1640 supplemented with L-glutamine, 10% fetal bovine serum (FBS; Gibco^®^, Milan, Italy), and 1% penicillin–streptomycin (Merck, Milan, Italy). The SH-SY5Y human neuroblastoma cell line (A.T.C.C., Manassas, VA, USA; passages 9–11) was cultured in 75 cm^2^ flasks in complete medium consisting of DMEM/F-12 (1:1) with L-glutamine, 10% FBS (Gibco^®^, Milan, Italy), and 1% penicillin–streptomycin. Both cell lines were maintained at 37 °C in a humidified atmosphere with 5% CO_2_ until reaching 70–80% confluence and were detached using an EDTA–trypsin solution (Merck). Experiments were conducted using cells derived from similar passage ranges to ensure experimental reliability. Cell counts were performed using a hemocytometer after trypan blue staining.

### 2.3. In Vitro Model of Microglial Stress-Correlated Neuroinflammation

BV2 cells were seeded in 6-well plates at a density of 3.0 × 10^5^ cells/well in complete RPMI 1640 medium containing 10% FBS. Upon reaching approximately 70% confluence, cells were stimulated with 100 nM CRH for 24 h [24]. Cells were treated with BPC or single constituents for 4 h prior to CRH stimulation. Unstimulated BV2 cells served as controls. At the end of the treatments, cell viability assays and biochemical analyses were performed.

### 2.4. In Vitro Model of Microglial Stress-Correlated Neurotoxicity

SH-SY5Y cells were exposed to conditioned medium from CRH-stimulated BV2 cells, with or without treatment with BPC or single constituents for 72 h. SH-SY5Y cells exposed to medium from unstimulated BV2 cells was used as control group. At the end of the treatments, cell viability assays and biochemical analyses were performed.

### 2.5. In Vitro Iron-Dependent Oxidative Stress Model of Neurotoxicity

SH-SY5Y cells were cultured as described above and incubated with iron(II) sulfate and L-ascorbic acid (Fe/Asc) at different concentrations (2.5/5 mM; 1.25/2.5 mM; 0.5/1 mM; 0.25/0.5 mM) for 24 h to induce a ferroptosis-like mechanism of neurotoxicity, as previously described [25].

### 2.6. Cell Morphology Analysis

At the end of cell stimulation and treatment, photos of the wells were taken by Leica DM IL LED FLUO optical microscope in dark-field mode and analyzed through the ImageJ software by experimenters blind to the cell culture conditions, as described in [26]. Briefly, for each image, the diameter of ten cells was measured to calculate the average cell length. The same procedure was used to determine the soma diameter. After that, the cell density was quantified as the number of cells per mm^2^ in at least ten randomly selected microscopic fields. The percentage of cells in a proinflammatory state was calculated. The proinflammatory phenotype was evaluated through morphological analysis, considering the presence of elongated cellular processes, increased soma size, and a shift from the rounded morphology typical of resting cells to spindle-shaped or multipolar forms indicative of microglial activation. For each image, the proportion of cells exhibiting proinflammatory characteristics was calculated relative to the total number of cells. The cell length was additionally measured using ImageJ software and expressed in micrometers to further discriminate inflammatory states, with non-proinflammatory cells measuring approximately 20 µm and proinflammatory cells exceeding 40 µm. The color variations qualitatively reflect differences in how cellular structures scatter light. Three independent experiments were performed for each treatment group.

### 2.7. SRB Test

BV2 cells were seeded in 96-well plates at a density of 2 × 10^4^ cells/well in 200 µL of complete medium. Cells were treated with the different concentrations of the compound and stimulated with 100 nM CRH for 24 h. After treatment, the medium was removed, and 100 µL of Hank’s Balanced Salt Solution (HBSS) and 25 µL of 50% trichloroacetic acid (TCA) were added to each well. Plates were incubated at 4 °C for 1 h. Subsequently, the wells were washed five times with 200 µL of double-distilled water, and plates were left to dry upside down overnight at room temperature. The following day, the cells were stained with 30 µL of sulforhodamine B (SRB; 4 mg/mL in 1% acetic acid) for 30 min in the dark, with plates placed on an orbital shaker. Excess dye was removed by washing four times with 1% acetic acid (200 µL/well). Finally, 200 µL of Tris-HCl buffer (pH 10) was added to each well, and the plates were shaken for 5 min. Absorbance was measured at 570 nm using a spectrophotometer (HiPo MPP-96; Biosan, Riga, Latvia). The cell viability was expressed as absorbance values proportional to the number of viable cells.

### 2.8. MTT Test

Cell viability and proliferation were assessed using the MTT assay, which measures mitochondrial metabolic activity. MTT (3-(4,5-dimethylthiazol-2-yl)-2,5-diphenyltetrazolium bromide) is a yellow tetrazolium salt that is reduced to insoluble purple formazan crystals by mitochondrial reductase enzymes in viable cells. SH-SY5Y cells were seeded in 96-well plates at a density of 2 × 10^4^ cells/well in 200 µL of medium and treated with the indicated concentrations of the compound. Cells were analyzed under basal conditions and in the presence of BV2 conditioned medium. After the appropriate incubation time, 20 µL of MTT solution (4 mg/mL in 1× PBS; 1:10 relative to the culture volume) was added to each well under light-protected conditions. Plates were incubated for 45 min at 37 °C, protected from light. Thereafter, the medium was carefully removed, and 100 µL of dimethyl sulfoxide (DMSO) was added to each well to dissolve the formazan crystals. The contents were pipetted several times to ensure complete solubilization, and 90 µL from each well was transferred to a new 96-well plate. Blank wells containing DMSO only were included. The absorbance was measured at 570 nm using a spectrophotometer (HiPo MPP-96; Biosan).

### 2.9. Western Blot Analysis

BV2 cells were lysed in lysis buffer, and insoluble material was removed by centrifugation at 12,000× *g* for 30 min at 4 °C. Protein concentration in the supernatant was determined using the Bradford assay (Merck, Milan, Italy). Equal amounts of protein (20 µg) were separated by 10% SDS–PAGE and transferred onto nitrocellulose membranes using a Trans-Blot Turbo Transfer Starter System (Bio-Rad Laboratories, Milan, Italy) [27]. Membranes were incubated overnight at 4 °C with the following primary antibodies: anti-Nrf2 (1:200; Santa Cruz Biotechnology, Dallas, TX, USA), anti-Sirt-1 (1:1000; Santa Cruz Biotechnology, sc-74465), p-JNK (Thr183/Tyr185) (1:750; Cell Signaling, Danvers, MA, USA, #4668), anti-AChE (1:500; Genetex GTX101648, Irvine, CA, USA), and anti-ChAT (1:500; Genetex GTX637915). After washing with PBS containing 0.1% Tween-20, membranes were incubated for 2 h at room temperature with horseradish peroxidase–conjugated goat anti-rabbit or anti-mouse secondary antibodies (1:3000; Jackson ImmunoResearch Labs, West Grove, PA, USA). Immunoreactive bands were visualized using an enhanced chemiluminescence detection system (ChemiDoc Imaging System; Bio-Rad, Milan, Italy). Band intensity, expressed as pixels/mm^2^, was quantified using ImageJ software (version 2.14), with exposure conditions kept constants for all samples. Protein expression levels were normalized to GAPDH protein content.

### 2.10. Statistical Analysis

Statistical analyses were performed using Student’s *t*-test or one-way analysis of variance (ANOVA), followed by Tukey’s post hoc tests, as appropriate. A *p* value < 0.05 was considered statistically significant. Data are presented as the mean ± standard error of the mean (SEM). All analyses were conducted using GraphPad Prism software (version 10.6.1; San Diego, CA, USA).

## 3. Results

### 3.1. Protection by Combined B. monnieri, Phosphatidylserine, and Choline (BPC) on CRH-Induced Microglia Activation

#### 3.1.1. CRH Stimulation of BV2 Microglia Cells

To reproduce a condition of stress-correlated neuroinflammation, we optimized an in vitro model in which BV2 microglia cells were stimulated with the corticotropin-releasing hormone (CRH).

**Figure 1:**
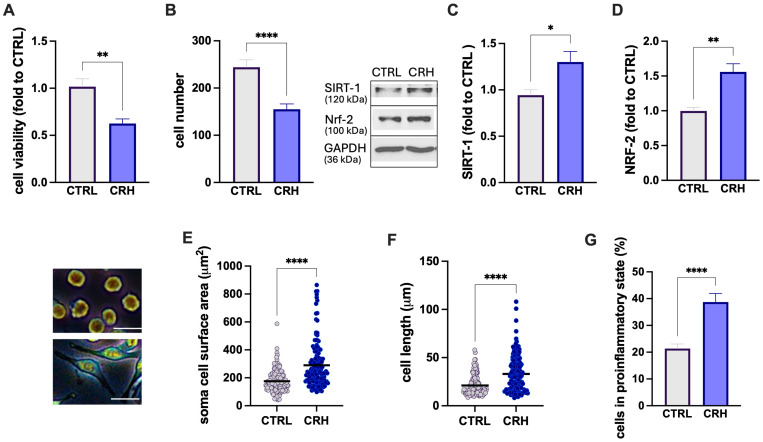
CRH-induced microglia activation. Decrease in cell viability (**A**) and cell number (**B**), increase in SIRT-1 (**C**) and Nrf-2 (**D**) protein expression, cell soma area (**E**), diameter (**F**), and number of cells in the proinflammatory state (**G**) by exposure of BV2 microglia cells to CRH 100 nM for 24 h. Scale bar: 20 µm. Vertical lines represent s.e.m.; * *p* < 0.05, ** *p* < 0.01, **** *p* < 0.0001.

Microglia are highly dynamic cells that can adopt distinct context-dependent phenotypes in response to environmental stimuli. These phenotypic states are commonly characterized by a combination of morphological features, molecular markers, and functional readouts parameter [28]. Accordingly, in our experimental design, cell morphology was analyzed in parallel with cell viability assays, cell number, and the expression of oxidative stress- and inflammation-related markers. Exposure of BV2 to CRH 100 nM for 24 h induced a proinflammatory phenotype characterized by a reduction in cell viability (Figure 1A) and cell number (Figure 1B). Microglia activation was characterized by an increased oxidative stress as revealed by the higher levels of antioxidant proteins SIRT1 (Figure 1C) and NRF-2 (Figure 1D). CRH also produced a cell morphology modification, as indicated by an increase in the cell soma area (Figure 1E) and diameter (Figure 1F). The induction of an overall proinflammatory phenotype was also demonstrated by the increased percentage of cells in the proinflammatory state (Figure 1F).

#### 3.1.2. Attenuation by BPC of CRH-Stimulated BV2 Cell Inflammatory Morphology

**Figure 2:**
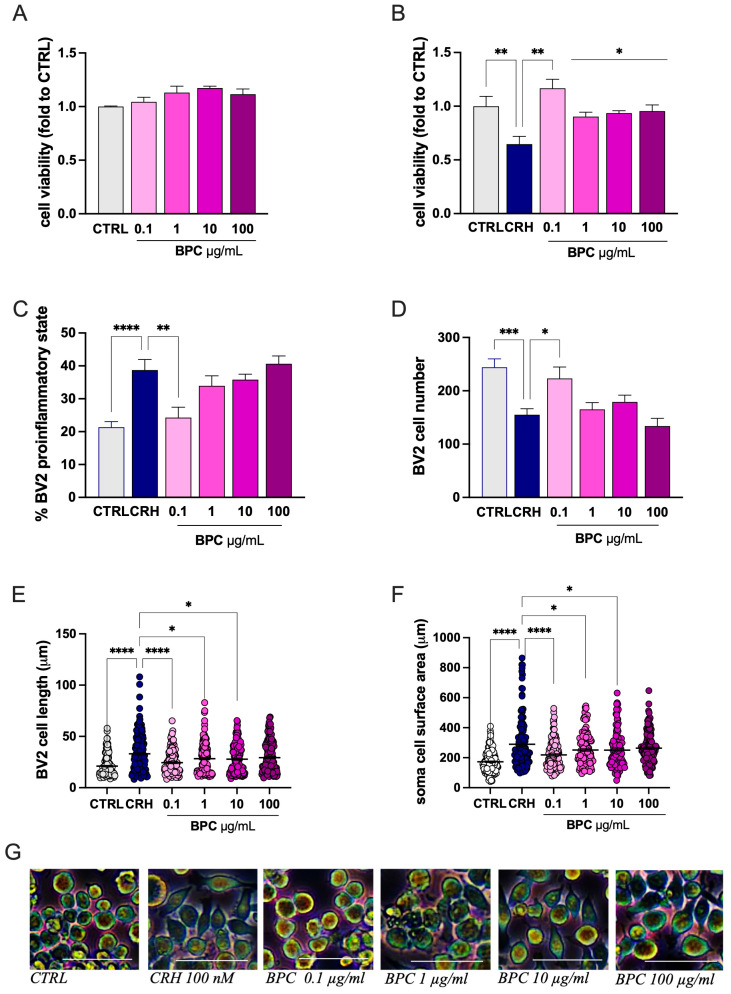
Attenuation by BPC of CRH-induced microglia proinflammatory morphology. (**A**) Lack of effect of BPC (0.1–100 µg/mL) on BV2 cell viability at resting conditions. (**B**) Reversal of CHR-induced reduction in BV2 cell viability by BPC. (**C**) BPC treatment reduction in the percentage of cells in the proinflammatory state previously increased by CRH stimulation. (**D**) Restoration by BPC of cell number reduced by CRH exposure. Reduction in the CRH-induced increase in cell diameter (**E**) and soma area (**F**). (**G**) Representative images of CRH-stimulated cells treated with BPC (0.1–100 µg/mL). Scale bar: 50 µm. BV2 microglia cells were exposed to CRH 100 nM for 24 h. Vertical lines represent s.e.m.; * *p* < 0.05, ** *p* < 0.01, *** *p* < 0.001, **** *p* < 0.0001.

**Figure 3:**
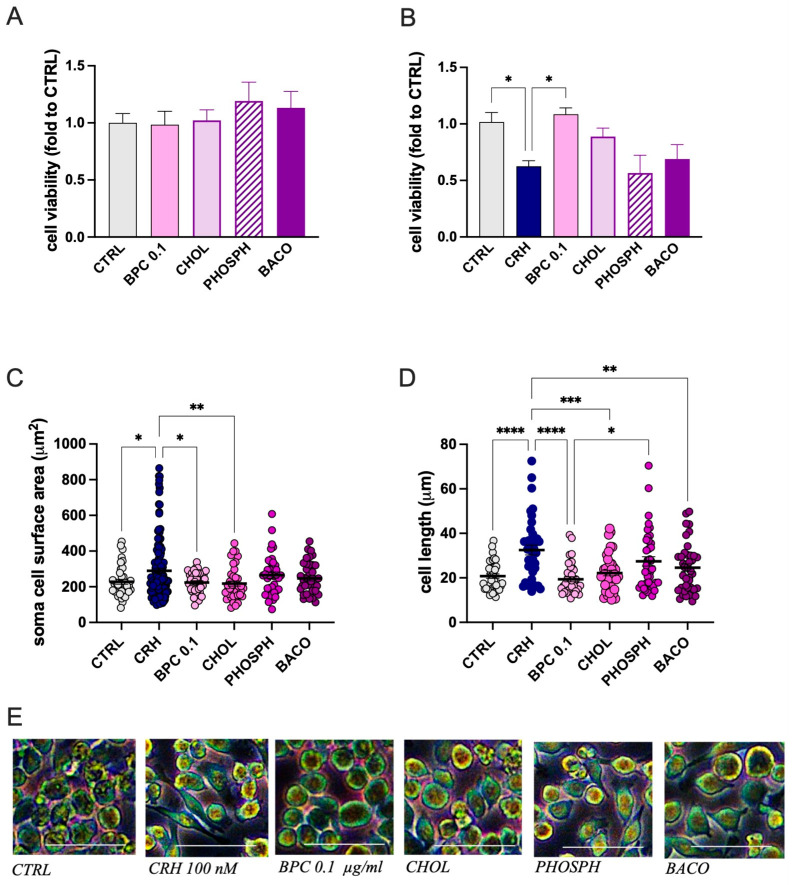
Effect of BPC constituents on CRH-stimulated BV2 cell morphology. (**A**) Lack of alteration in cell viability by BPC, CHOL, PHOSPH, and BACO. (**B**) Partial reversal of CRH-induced reduction in cell viability by CHOL. (**C**) Reduction in CRH-induced increase in soma area by CHOL. (**D**) Reduction in cell diameter by CHOL, PHOSP, and BACO. (**E**) Representative images of BV2 cells, Scale bar: 50 µm. BV2 microglia cells were exposed to CRH 100 nM for 24 h. Vertical lines represent s.e.m.; * *p* < 0.05, ** *p* < 0.01, *** *p* < 0.001, **** *p* < 0.0001. BPC 0.1 µg/mL, CHOL 3.6 ng/mL, PHOSPH 2 ng/mL, BACO 1.25 ng/mL.

Under resting conditions, combined *B. monnieri*, phosphatidylserine, and choline (BPC) at all concentrations tested (0.1–100 µg/mL) did not alter the cell viability compared with the CTRL group, indicating the absence of toxicity (Figure 2A). CRH exposure reduced the cell viability by approximately 40%, which was completely reversed by BPC 0.1 µg/mL. Higher doses produced a partial reversal of CRH-induced toxicity (Figure 2B). BCP returned to basal levels the increased percentage of cell in the proinflammatory state (Figure 2C) and the reduced cell number (Figure 2D) following CRH stimulation at the dose of 0.1 µg/mL, while the protective effect gradually disappeared at higher doses. A comparable profile was observed for morphological analysis. The CRH-induced increase in the cell diameter (Figure 3E) and soma area (Figure 2F) was reversed by BPC 0.1 µg/mL. The effect slowly diminished by increasing the doses. On this basis, the dose of BPC 0.1 µg/mL was chosen for further studies.

#### 3.1.3. Role of Constituents on CRH-Stimulated BV2 Cell Morphology

The positive results obtained with the evaluation of BPC activity encouraged us to investigate the effect of single constituents to assess the presence of an additive or synergistic interaction. Evaluation of the effect on cell viability produced by choline (CHOL), phosphatidylserine (PHOSPH), and *B. monnieri* L. extract (BACO) examined at the concentration present in BPC 0.1 µg/mL showed a lack of any toxic effect at resting conditions (Figure 3A). After CRH stimulation, the reduction in cell viability was partially reversed by CHOL compared with BPC, while PHOSPH and BACO were devoid of any effect (Figure 3B). Similarly, the increase in the cell diameter (Figure 3C) and soma area (Figure 3D) were significantly reduced by CHOL. PHOSPH and BACO were ineffective.

#### 3.1.4. Modulation of Stress-Related Markers

**Figure 4:**
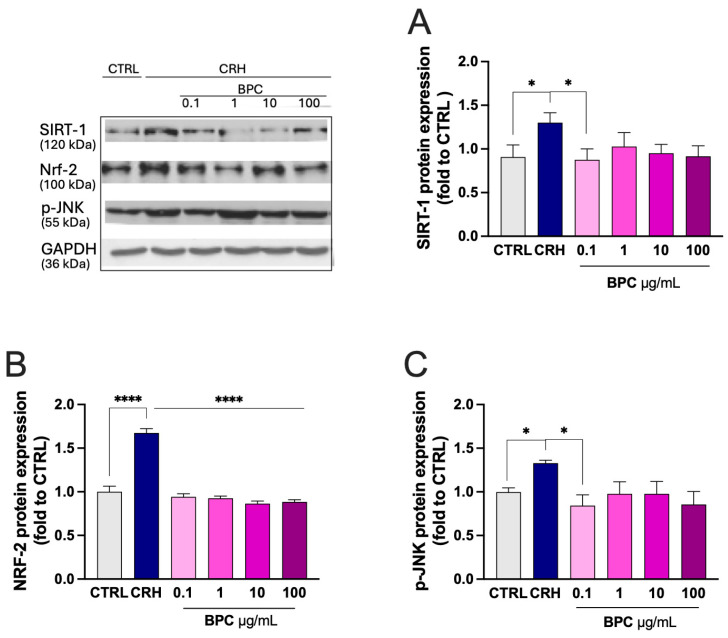
Attenuation by BPC of CRH-induced stress-related markers. CRH exposure increase in SIRT-1 (**A**), Nrf-2 (**B**), and p-JNK (**C**) levels and restoration of basal levels by BPC (0.1–100 µg/mL). BV2 microglia cells were exposed to CRH 100 nM for 24 h. Vertical lines represent s.e.m.; * *p* < 0.05 **** *p* < 0.0001.

To better define the efficacy of BPC, the effect of treatment on the main stress-related markers was investigated. CHR-stimulated BV2 cells showed increased expression of the antioxidant enzymes SIRT-1 (Figure 4A) and NRF-2 (Figure 4B). Furthermore, increased levels of the phosphorylate form of the stress-associated MAPK JNK were produced (Figure 4C). BPC 0.1–100 µg/mL reversed the CRH-induced effect by returning the protein levels to the control values.

### 3.2. Protection by BPC from Neurotoxicity

#### 3.2.1. Effect of BPC on CRH-Induced Neurotoxicity on SH-SY5Y Cells

**Figure 5:**
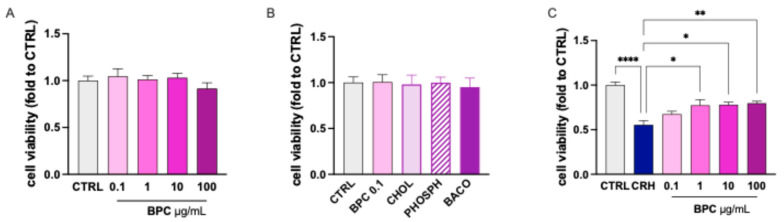
Attenuation by BPC of CRH-induced neurotoxicity. Lack of alteration of SH-SY5Y cell viability by BPC (**A**) and single constituents (**B**) at basal conditions. (**C**) Reduction in cell viability by CRH-stimulated BV2 conditioned medium and dose-dependent attenuation by BPC (0.1–100 µg/mL). Vertical lines represent s.e.m.; * *p* < 0.05, ** *p* < 0.01, **** *p* < 0.0001.

To evaluate the capability of BPC to protect from stress-related neurotoxicity, the effect of treatment was investigated on SH-SY5Y cells. Determination of the cell viability at resting conditions showed the lack of any toxic effect of BPC at doses ranging from 0.1 to 100 µg/mL (Figure 5A). Similar results were obtained following treatment of cells with individual constituents at doses present in BPC 0.1 µg/mL (Figure 5B). To determine whether BPC was able to produce neuroprotective effects in the presence of stress-induced neuroinflammation, SH-SY5Y cells were exposed to conditioned medium collected from BV2 microglial cells stimulated with CRH 100 nM in the absence or presence of BPC. The CRH-induced reduction in cell viability was dose-dependently attenuated by BPC (0.1–100 µg/mL; Figure 5C).

#### 3.2.2. Effect of BPC on Cholinergic System

**Figure 6:**
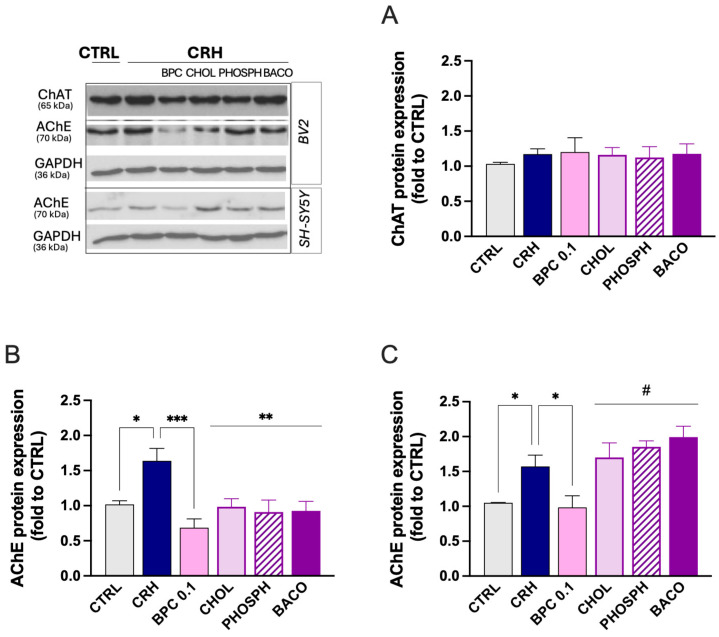
BPC activation of the cholinergic system. (**A**) Lack of effect of CRH stimulation, BCP, CHOL, PHOSPH, and BACO on ChAT protein levels in BV2 cells. (**B**) Increase in AChE protein levels in BV2 cells by CRH exposure and prevention by BCP, CHOL, PHOSPH, and BACO. (**C**) AChE protein increased expression by exposure of SH-SY5Y cells to CRH-stimulated BV2 cells conditioned medium and effect of BCP, CHOL, PHOSPH and BACO treatment. Vertical lines represent s.e.m.; * *p* < 0.05, ** *p* < 0.01, *** *p* < 0.001, # *p* < 0.05 vs. BPC 0.1. CRH 100 nM for 24 h. BPC 0.1 µg/mL, CHOL 3.6 ng/mL, PHOSPH 2 ng/mL, BACO 1.25 ng/mL.

To correlate the neuroprotective effect of BCP with a beneficial effect on memory processes, we evaluated the capability of the combination to activate the cholinergic system. We first investigated the expression of the enzymes involved in the synthesis and degradation of acetylcholine (Ach) in the model of neuroinflammation. Stimulation of BV2 cells with CHR did not alter the expression of the choline acetyl transferase (ChAT), the enzyme responsible for the synthesis of Ach, and pretreatment with BPC 0.1 µg/mL or single constituents did not produce any significant effect (Figure 6A). Conversely, in the same experimental conditions, an increase in the levels of acetylcholinesterase (AChE), the enzyme responsible for the hydrolysis of Ach, were increased. BCP 0.1 µg/mL drastically reduced the AChE protein expression, and similar effects were observed for all single constituents (Figure 6B). Then, to further assess the involvement of the cholinergic system in the neuroprotective activity of BPC, SH-SY5Y cells were exposed to the conditioned medium from CRH-stimulated BV2 cells. Consistently with the effects produced on microglia cells, stimulated SH-SY5Y cells showed an increased expression of AChE protein levels that was completely abolished by BPC 0.1 µg/mL. Single constituents were all ineffective (Figure 6C).

#### 3.2.3. Effect of BPC on Neurotoxicity Induced by Fe/Asc Exposure

**Figure 7:**
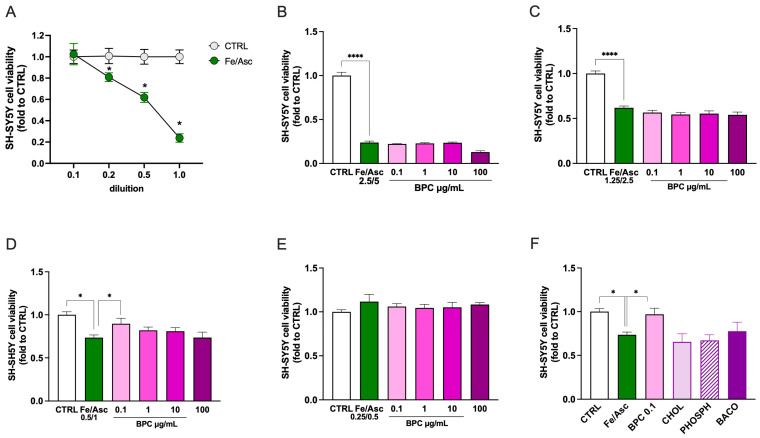
Neuroprotective activity of BPC against iron(II) sulfate (Fe)/L-ascorbic acid (Asc) toxicity in SH-SY5Y cells. (**A**) Dose-dependent reduction in cell viability of SH-SY5Y cells exposed to Fe/Asc 2.5 and 5 mM, respectively, (indicated as 1) and dilutions for 24 h. Cell viability following exposure to Fe/Asc 2.5/5 mM (**B**), 1.25/2.5 mM (**C**), 0.5/1 mM (**D**), and 0.25/0.5 mM (**E**) and treatment with BPC (0.1–100 µg/mL). (**F**) Effect of BPC (0.1 µg/mL), CHOL (3.6 ng/mL), PHOSPH (2 ng/mL), and BACO (1.25 ng/mL) on neurotoxicity induced by Fe/Asc 0.5/1 mM. * *p* < 0.05, **** *p* < 0.001.

To further validate the efficacy of BPC as a neuroprotective agent, an iron-dependent oxidative stress model of neurotoxicity consistent with ferroptosis-like mechanisms was employed. SH-SH5Y neuroblastoma cells were exposed to iron(II) sulfate and L-ascorbic acid (Fe/Asc) at different concentrations. A dose-dependent reduction in cell viability was observed, showing a lack of effect at Fe/Asc 0.25/0.5 mM, mild neurotoxicity at 0.5/1 mM, moderate neurotoxicity at 1.25/2.5 mM, and severe neurotoxicity at 2.5/5 mM (Figure 7A). In each of these experimental conditions, dose–response curves for BPC (0.1–100 µg/mL) were performed. Treatment was ineffective when SH-SY5Y cells were exposed to an Fe 2.5/Asc 5 (Figure 7B) or Fe 1.25/Asc 2.5 (Figure 7C) stimulus. Conversely, the reduction in cell viability induced by stimulation with Fe 0.5/Asc 1 was significantly reversed by BPC 0.1 µg/mL (Figure 7D). No effect was produced by treatment at a lower concentration of Fe/Asc, which was unable to alter cell viability (Figure 7E). Investigation into the activity of single constituents present in BPC 0.1 µg/mL exposed to Fe 0.5/Asc 1 showed the lack of significant effects by every treatment (Figure 7F).

#### 3.2.4. Restoration of SIRT-1 by BPC After Fe/Asc Exposure

**Figure 8:**
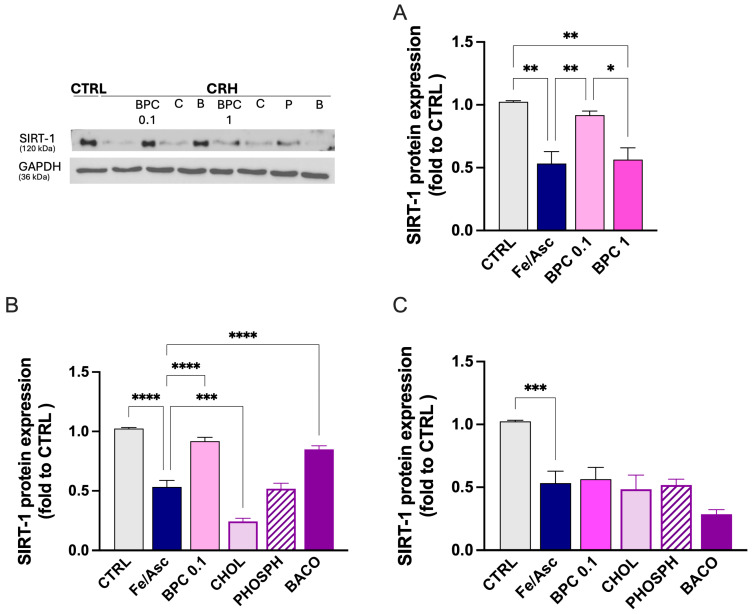
BPC restoration of SIRT-1 after Fe/Asc exposure. (**A**) Effect of BPC 0.1 and 1 µg/mL on SIRT-1 protein levels in SH-SY5Y cells exposed to Fe/Asc 0.5 and 1 mM, respectively. (**B**) Effect of BPC (0.1 µg/mL), CHOL (3.6 ng/mL), PHOSPH (2 ng/mL), and BACO (1.25 ng/mL) on neurotoxicity induced by Fe/Asc 0.5/1 mM. (**C**) Effect of BPC (1 µg/mL), CHOL (36 ng/mL), PHOSPH (20 ng/mL), and BACO (12.5 ng/mL) on neurotoxicity induced by Fe/Asc 0.5/1 mM. * *p* < 0.05, ** *p* < 0.01, *** *p* < 0.001, **** *p* < 0.001.

To further assess the neuroprotective efficacy of BPC and single constituents, the effect of treatment with BPC 0.1 and 1 µg/mL was tested after Fe 0.5/Asc 1 stimulus application. Exposed SH-SY5Y cells showed a reduced expression of SIRT1 protein levels that were completely restored by BPC 0.1, while BPC 1 was ineffective (Figure 8A), consistently with the cell viability results. By investigating the role played by single constituents, BACO contained in BPC 0.1 µg/mL increased SIRT1 levels, while CHOL and PHOSP were ineffective (Figure 8B). A lack of activity for all constituents at the concentration present in BPC 1 µg/mL was observed (Figure 8C).

## 4. Discussion

The present study investigated the efficacy of a therapeutic approach aimed at modulating stress-altered molecular targets within the central nervous system using cell-based experimental models. Specifically, the effects of a combination of *B. monnieri*, phosphatidylserine, and choline (BPC) were evaluated in an in vitro model of stress-related neuroinflammation induced by corticotropin-releasing hormone (CRH). The study was designed to assess the cellular neuroprotective, anti-inflammatory, and antioxidant properties of BPC under controlled experimental conditions using BV2 microglial cells and SH-SY5Y neuronal cells, rather than to model complex behavioral or cognitive outcomes.

Prolonged activation of stress pathways is known to disrupt immune regulation and promote neuroinflammatory processes. In line with this evidence, we optimized a CRH-based in vitro model of stress-correlated microglial activation. The validity of the experimental system was confirmed by demonstrating that CRH treatment induced a proinflammatory phenotype in BV2 microglial cells, consistent with previous reports describing CRH-mediated microglial activation [24,29]. Moreover, CRH exposure led to the increased expression of antioxidant-related proteins SIRT-1 and NRF-2, suggesting the activation of endogenous protective responses to oxidative stress [30]. These findings are consistent with previous studies showing that stress-induced microglial activation is accompanied by the engagement of antioxidant signaling pathways, including NRF-2 and SIRT-1 [31,32].

Neuroinflammation is widely recognized as a cellular mechanism contributing to neuronal vulnerability. In our experimental model, CRH-induced microglial activation resulted in increased oxidative stress and inflammatory signaling, which translated into reduced neuronal viability in SH-SY5Y cells exposed to conditioned medium from stimulated BV2 microglia. These observations align with established evidence indicating that soluble factors released by activated microglia can impair neuronal survival in vitro [33,34]. Collectively, these results confirm that the adopted cellular model effectively reproduces stress-related neuroinflammatory and neurotoxic conditions at the cellular level.

Growing evidence supports the use of adaptogenic and neuroactive compounds to counteract stress-associated cellular alterations. Natural products such as *Withania somnifera*, *Panax ginseng*, and *Rhodiola rosea* have been extensively studied for their antistress properties [12]. *B. monnieri*, in particular, is well recognized for its antioxidant and neuroprotective activities, largely attributed to its bacoside content. Previous studies have demonstrated that *Bacopa* enhances neuronal resistance to oxidative damage in vitro [35], while clinical studies have reported improvements in cognitive performance under specific conditions [17,19]. However, such clinical observations cannot be directly extrapolated from the present cellular findings.

In the current study, *B. monnieri* was evaluated in combination with choline and phosphatidylserine. Choline is a precursor of acetylcholine and plays an essential role in cellular membrane integrity and neurotransmitter metabolism, while also contributing to the modulation of inflammatory responses [36]. Prenatal choline supplementation has been shown to accelerate long-term memory development in animal models [37]. Phosphatidylserine is a key structural component of neuronal membranes and is involved in synaptic function and cellular stress responses [20,38]. The combination was selected to investigate whether these compounds could exert complementary protective effects at the cellular level.

Furthermore, BPC treatment restored the oxidative balance, as indicated by normalization of SIRT-1 and NRF-2 expression and attenuation of stress-related JNK MAPK hyperphosphorylation. These data support the notion that the protective effects of BPC are mediated, at least in part, through reinforcement of endogenous antioxidant defenses [39,40]. The antioxidant effects of *B. monnieri* are well documented in the literature, supporting its therapeutic potential in protecting the brain from oxidative damage [41,42]. Additionally, emerging evidence highlights choline as a potential modulator of oxidative stress and reactive oxygen species (ROS) production [43,44]. These data support the notion that the protective effects of BPC are mediated, at least in part, through reinforcement of endogenous antioxidant defenses.

Attenuation of microglial inflammatory signaling was associated with neuroprotective effects in SH-SY5Y neuronal cells. Exposure to conditioned medium from CRH-stimulated BV2 cells reduced neuronal viability, an effect that was significantly mitigated by BPC pretreatment. Additionally, BPC was protective in an iron-dependent oxidative stress model consistent with ferroptosis-like mechanisms. Ferroptosis is a regulated form of necrotic cell death driven by iron-dependent lipid peroxidation and has been implicated in several neurodegenerative disorders [45,46]. Our findings suggest that BPC may act as a potential adjunctive strategy in early stages of neuronal damage. Notably, under mild neuronal stress conditions, BPC enhanced cell survival and improved resistance to oxidative insults. While ferroptosis has been implicated in neurodegenerative disorders, the present findings should be interpreted as evidence of cellular resistance to oxidative injury, rather than as direct indications of disease-modifying activity.

The cholinergic system plays a crucial role in regulating cognitive and emotional responses to stress [47]. Dysregulation of cholinergic signaling has been associated with neurodegenerative diseases, while chronic microglial activation contributes to sustained neuroinflammation and cognitive decline [48]. In this context, we investigated the expression of acetylcholinesterase (AChE) and choline acetyltransferase (ChAT), two key enzymes involved in cholinergic neurotransmission. In agreement with previous studies, inflammatory stimulation increased AChE expression without significantly affecting the ChAT levels [49], our model revealed elevated AChE levels with unchanged ChAT expression. Importantly, BPC treatment significantly attenuated AChE upregulation in both BV2 microglia and SH-SY5Y cells exposed to CRH-conditioned medium, suggesting a protective effect on cholinergic-related cellular alterations induced by stress-like conditions. While dysregulation of cholinergic signaling has been associated with cognitive impairment, the present findings are limited to molecular and enzymatic changes observed in vitro. Further studies will be required to investigate whether BPC influences additional cholinergic components or receptor signaling in more complex in vivo systems.

Notably, BPC was more effective in modulating the cholinergic system than its individual components, suggesting a synergistic interaction between *B. monnieri* and choline. Both compounds are known to influence cholinergic signaling, with Bacopa acting as an AChE inhibitor and choline promoting acetylcholine synthesis and ChAT activity [50]. Analysis of the individual components further confirmed that the BPC combination was more effective in counteracting oxidative stress than single treatments. While individual compounds were unable to fully prevent CRH-induced inflammatory alterations, their combination exerted a stronger protective effect. This enhanced efficacy likely arises from parallel modulation by the three constituents of complementary cellular processes, including attenuation of microglial inflammatory activation, reinforcement of endogenous antioxidant defenses, and regulation of cholinergic-related molecular markers. Nevertheless, the present study does not address pharmacokinetics, blood–brain barrier penetration, or behavioral outcomes, which remain essential aspects for future investigation.

## 5. Conclusions

In conclusion, this study demonstrates that the BPC combination exerts significant anti-inflammatory, antioxidant, and neuroprotective effects in cellular models of stress-induced neuroinflammation and oxidative injury. Notably, the results consistently demonstrate that the combined formulation exerts superior efficacy compared with the individual components administered alone. These findings provide mechanistic insights into how BPC modulates stress-related cellular pathways by concurrently attenuating neuroinflammation and oxidative stress, while influencing cholinergic-related molecular markers. While these results should be interpreted with caution due to the inherent limitations of in vitro experimental models, they underscore the potential of BPC to promote a protective cellular environment under conditions of stress. Although the data support the potential of BPC to counteract molecular processes associated with neuronal vulnerability, future studies employing additional cell lines and animal models will be required to validate and extend our findings and to confirm their relevance for cognitive function. Future investigations will be necessary to assess the translational significance of these cellular observations.

## Data Availability

The data presented in this study are available on request from the corresponding author.

## Abbreviations

The following abbreviations are used in this manuscript:

Ach	acetylcholine
AChE	acetylcholinesterase
BBB	blood brain barrier
BPC	*Bacopa monnieri*, phosphatidylserine, and choline
ChAT	choline acetyltransferase
CNS	central nervous system
CRH	corticotropin-releasing hormone
HPA	hypothalamic–pituitary–adrenal
SNS	sympathetic nervous system
SRB	sulforhodamine B
